# The microbiome of pseudomyxoma peritonei: a scoping review

**DOI:** 10.1515/pp-2024-0016

**Published:** 2025-05-22

**Authors:** D. Sara Portela, Anshini Jain, Michael Flood, Aonghus Lavelle, Glen Guerra, Meera Patel, Omer Aziz, Satish Warrier, Alexander Heriot, Helen Mohan

**Affiliations:** Peter MacCallum, Melbourne, Australia; University College Cork, Cork, Ireland; The Christie, Manchester, UK

**Keywords:** pseudomyxoma peritonei, peritoneal cancer, cytoreductive surgery, hyperthermic intraperitoneal chemotherapy, microbiome, microbiota

## Abstract

There is growing interest in the role of the microbiome in carcinogenesis, but few studies examine the microbiome of pseudomyxoma peritonei (PMP). This scoping review summarises the microorganisms identified in PMP samples and examines the evidence of their role in disease outcomes. The methodology was developed in accordance with the PRISMA-ScR framework and checklist. Nine relevant studies were included. Microbiological testing was performed on PMP samples from 85 patients. At the phylum level, Proteobacteria was detected in greatest relative abundance in tumour tissue, cellular and acellular mucin. The relative proportion of different phyla more closely resembled the gut microbiome in inflammatory bowel disease than in a healthy gut. High-grade specimens showed significantly higher bacterial density than low-grade specimens and non-neoplastic non-perforated appendix specimens. Survival data of 58 patients were published, correlating outcomes to pre-operative antibiotic administration. Observed differences were not statistically significant. There is evidence of an altered bacterial profile in PMP samples compared to a healthy gut microbiome, the significance of which is unclear. Significant methodological challenges remain in this field of study. This scoping review supports the need for further analysis of the PMP bacterial profile, using methodologies that incorporate controls and deliver taxonomic resolution at species level.

## Introduction

### Rationale

Pseudomyxoma peritonei (PMP) is a rare phenomenon characterised by the progressive accumulation of mucin within the peritoneum. Although PMP has been associated with mucinous tumours of different origins [1], 2], the majority of cases results from a ruptured appendiceal mucinous neoplasm [3]. The tumour cells that are released into the abdominal cavity continue to proliferate and exude mucin, resulting in mucinous ascites and tissue deposits, which can develop insidiously over time. As the volume of mucin increases, so does the intra-abdominal pressure, leading to the compression of adjacent structures and eventually bowel obstruction [4].

Despite only affecting two to four per million annually [5], 6], the prognosis can be poor, depending largely on the histological subtype [7], 8]. Low-grade PMP [9], previously known as disseminated peritoneal adenomucinosis, has five-year survival rates of 84 %. On the other hand, high-grade PMP, previously known as peritoneal mucinous carcinomatosis, is associated with a 10–40 % five-year survival rate [10], 11].

Cytoreductive surgery (CRS) and hyperthermic intraperitoneal chemotherapy (HIPEC) is the mainstay treatment for PMP. However, the rate of surgical complications and associated morbidity is as high as 24–50 % and the recurrence rate is 25 % [7], 12], 13]. Given the paucity of treatment options for patients with PMP, new insights into the pathophysiology of the disease are essential to explore alternative potential therapeutic avenues.

The gut microbiome (the collective microbiota found within the gastrointestinal tract) has been linked to chronic diseases such as type 2 diabetes, hypertension and inflammatory bowel disease [14], and there is a growing interest in the role of pathogens in carcinogenesis and tumour progression [15], [16], [17]. Several pathogens including *Fusobacterium nucleatum* [18], *Peptostreptococcus anaerobius* [19], *Bacteroides fragilis* [20] and *Escherichia coli* [21], for example, are thought to contribute to carcinogenesis in colorectal cancer through a variety of molecular mechanisms [22]. Conversely, a growing body of evidence supports the protective effects of bacteria, particularly of healthy commensal gut microbiota, against inflammation and cancer. Short-chain fatty acids produced by microbial fermentation of dietary fibre not only have anti-inflammatory properties but also facilitate apoptosis of colonic malignant cells by inhibiting histone deacetylases [23]. The commensal microbiome of the digestive system also preserves the epithelial integrity and prevents pathogen overgrowth, reducing inflammation and colitis-associated cancers [24].

In PMP, one theory is that bacteria translocate from the intestinal lumen to the peritoneum at the time of appendiceal perforation, but few studies have examined the microorganisms that colonise PMP tissue.

### Objectives

A scoping review was carried out to systematically collect and synthesise the research done in this area and map it to the framework detailed above, to emphasise the importance of this topic and to highlight the current gaps in knowledge.

The aims of this scoping review were to determine what micro-organisms have been identified in PMP samples and explore what role, if any, they may play in disease outcomes.

## Methods

This scoping review was performed in accordance with the preferred reporting items for systematic reviews and meta-analyses extension for scoping reviews (PRISMA-ScR) framework and checklist [25]. This methodology was elected over a systematic review due to the paucity of existing data surrounding this emerging topic, which has only relatively recently gained traction in the scientific community. The final protocol was registered retrospectively with the Open Science Framework on the 7th March 2024 (DOI:10.17605/OSF.IO/WYE27).

### Eligibility criteria

Our search aimed to identify peer-reviewed journal papers that examined microorganisms collected from human PMP tissue or mucin samples. PMP from all reported tumour origins were included to maximise the search results. All study types were included given the highly specialised area of research, to allow a comprehensive review of all existing evidence. Papers outside of the scope of the research question were excluded. Review articles that did not provide novel data were also excluded.

### Information sources

PubMed, EMBASE and Scopus databases were used to identify potentially relevant studies.

### Search

**Figure 1:**
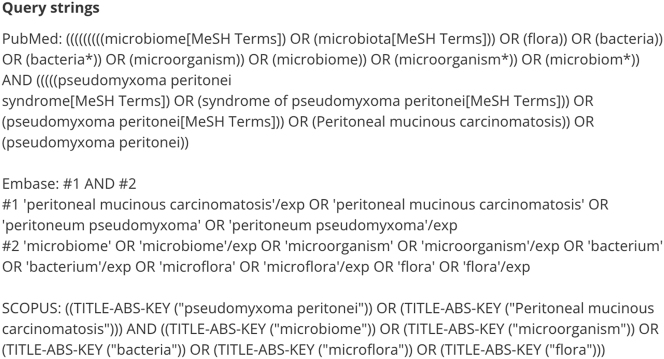
Search strategies used in PubMed, Embase and Scopus.

An initial search was performed to identify common keywords (e.g., pseudomyxoma peritonei, peritoneal cancer, microbiome, microbiota, *Pseudomonas*, *H. pylori*, cytoreductive surgery, mucin). Keywords outside the scope of our research question (e.g., cytoreductive surgery, mucin) were excluded to ensure focused results. Other keywords such as ‘PMP’ ‘peritoneal cancer’, ‘appendiceal cancer’, ‘peritoneal neoplasms’, ‘*Pseudomonas*’ and ‘*H. pylori’* were also excluded from the search as they lacked specificity and were likely to generate a large volume of insignificant results. Synonyms, truncations and MeSH terms were used in the PubMed search (Figure 1).

### Selection of sources of evidence

All search results were exported to Rayyan [26]. Duplicate results were removed. Titles and abstracts were assessed to determine relevance, according to the eligibility criteria described above. All studies that both histologically confirmed a diagnosis of PMP and performed microbiological analysis of the tumour tissue or mucin were selected for further screening to confirm relevance. Studies investigating the aetiology of infection after CRS/HIPEC in patients with PMP, for example, were excluded. Additionally, all animal studies were excluded. Two reviewers performed the screening process sequentially to identify relevant publications and reduce selection bias. Disagreements were discussed and resolved.

### Data charting process

Two reviewers independently extracted relevant data from all the selected manuscripts. The data was manually extracted and subsequently charted in an excel spreadsheet containing headings for each selected variable. The results tables generated by each reviewer were compared to identify and resolve any disagreements, and then merged.

### Data items

The variables recorded included: manuscript title; authors; year of publication; study population (i.e. primary site of PMP, histological grading); type of specimen collected (i.e. tissue vs. mucin); methodology (i.e. detection methods, taxonomic rank); main results; and limitations of the study.

### Synthesis of results

A two-stage analysis was performed. The primary analysis involved the identification and documentation of all microorganisms identified from PMP tissue and mucin samples, at the various taxonomic ranks. Subsequently, the literature was further examined to determine the possible clinical significance of the identified microorganisms.

## Results

### Selection of sources of evidence

**Figure 2:**
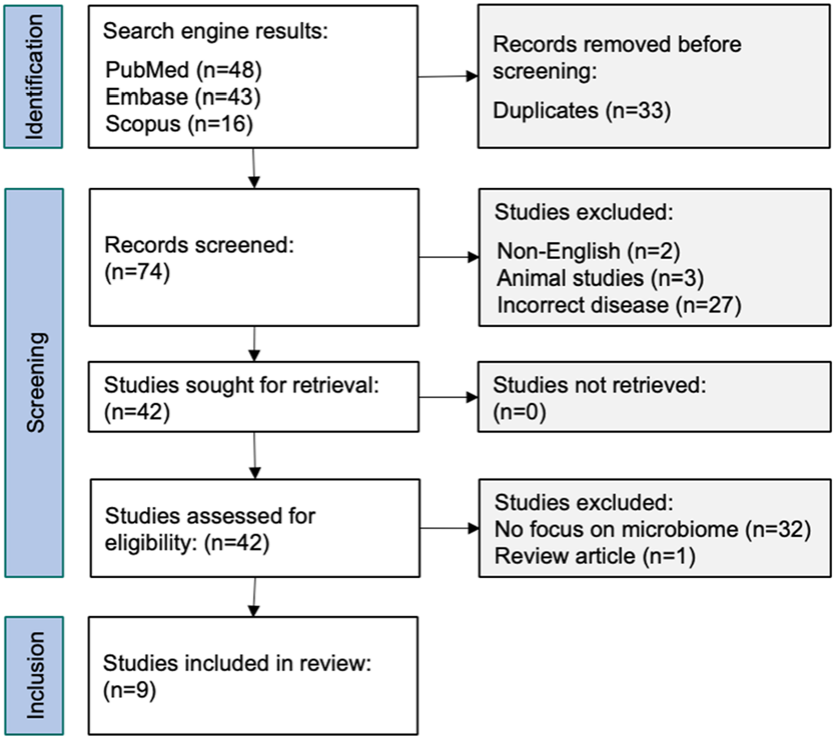
Database screening process and study selection.

A total of 107 results were generated from the searches. PubMed generated 48 results, Embase generated 43 and Scopus generated 16. Duplicates were identified and removed. The remaining 74 unique studies were assessed to determine relevance. After excluding all studies that did not satisfy our eligibility criteria, nine were selected for review (Figure 2).

### Characteristics of sources of evidence

Eight studies isolated, identified, and characterised microorganisms from PMP specimens collected intra-operatively (Table 1). There was significant heterogeneity in the type of study and methodology used. The relevant studies included two case reports, an announcement, two retrospective laboratory studies performing analysis on archived specimens, and three prospective laboratory studies. The microbial detection methods also varied amongst the different studies, including traditional culturing, *in-situ* hybridisation (ISH), V6 polymerase chain reaction (PCR) and whole genome sequencing.

**Table 1:** Summary of studies that have identifying microorganisms from PMP specimens.

Title	Author	Year	Study type	Cohort size	PMP origin	Histology	Specimen collected	Evidence of contamination	Microbiome control	Laboratorial control	Microbial detection methods used	Organisms identified
Low grade peritoneal mucinous carcinomatosis associated with human papilloma virus infection: case report	Gatalica et al.	2008	Case report	n=1	Cervix	Low-grade PMP (n=1)	Tissue specimens and mucinous ascites fluid collected at the time of CRS and HIPEC	No tumour was found in the contralateral ovary or appendix. Hysterectomy for a well-differentiated cervical mucinous adenocarcinoma eight year prior	None	Unknown	Tissue: ISH – probe with affinity to HPV genotypes 16, 18, 31, 33, 35, 39, 51, 52, 56, 58, and 66. Mucinous ascites: hybrid capture methodology	High-risk HPV sequences
A novel member of Chitinophagaceae isolated from a human peritoneal tumour	Lo et al.	2015	Announcement	n=1	Unknown	Low-grade PMP (n=1)	Unclear type of specimen, collected at the time of CRS and HIPEC.	No comment	None	Unknown	16S rRNA gene sequencing. Whole-genome sequencing	Strain PMP191F
*Parapseudoflavitalea muciniphila* gen. nov., sp. nov., a member of the family Chitinophagaceae isolated from a human peritoneal tumour and reclassification of *Pseudobacter ginsenosidimutans* as *Pseudoflavitalea ginsenosidimutans* comb. Nov	Lawson et al.	2020	Case report								16S rRNA gene sequencing. Whole genome sequencing performed. Cell morphology observed using phase-contrast microscope	Strain PMP191FT, which represents a novel species and genus for which the name *P. muciniphila* gen. nov., sp. nov. is proposed
Pseudomyxoma peritonei: is disease progression related to microbial agents? A study of bacteria, MUC2 and MUC5AC expression in disseminated peritoneal adenomucinosis and peritoneal mucinous carcinomatosis	Semino-mora et al.	2008	Retrospective laboratory study	n=16	Unknown	Low-grade PMP (n=6), high-grade PMP (n=10)	Tissue specimens collected at the time of CRS and HIPEC	No patients had signs of intra-abdominal sepsis (abdominal tenderness, fever, increased white blood cells count) or evidence of intestinal perforation.	Non-inflamed, unperforated, non-neoplastic appendices from (n=5) patients without PMP (resected at the time of gynaecological procedures)	Control for nonspecific binding	ISH – probe hybridizing to 16S rRNA sequence conserved among 19,973 typed and nonculturable bacteria; probe specific for *H. pylori* 16s rRNA and virulence factor cag. Fluorescence *in situ* hybridization (FISH) recognizing *H. pylori* 16S rRNA and cagA	Enteric bacteria were detected in all specimens. *H. pylori* was detected in all patients, but the density was lower than for TNCB
Under the hood: understanding the features of mucin in pseudomyxoma peritonei	Villarejo-Campos et al.	2023	Prospective laboratory study	n=6	Appendix (n=5), colon (n=1)	Low-grade PMP (n=3), acellular mucin (n=3)	Mucin samples collected at the time of CRS and HIPEC	No comment	None	Unknown	16S rRNA gene sequencing	Phylum: Proteobacteria, Actinobacteria, order: Pseudomonadales, Genus: *Pesudomonas*. Species: *Pseudomonas plecoglossicida*
A core microbiome associated with the peritoneal tumours of pseudomyxoma peritonei	Gilbreath et al.	2013	Retrospective laboratory study	n=11	Unknown	Unknown	Tissue specimens and mucin samples previously collected peri-operatively	No comment	None	No template control; control hybridisations for nonspecific binding	V6 PCR and sequencing. ISH: 16S and 23S rRNA-specific probes used for Actinobacteria, Bacteroidetes, Betaproteobacteria, Gammaproteobacteria, Firmicutes, Rhizobiales, Propionibacterium, Pseudomonas, Streptococcus and Verrucomicrobiales. Culturing and identification of bacterial isolates from PMP tissue	Phylum: Proteobacteria, Actinobacteria, Firmicutes, Bacteriodetes, Verrucomicrobia, Acidobacteria. Order: Verrucomicrobiales, Rhizobiales. Genus: *Methylobacterium*, *Variovorax*, *Escherichia_Shigella*, *Acinetobacter*, *Pseudomonas*, *Propionibacterium*, *Streptococcus*, *Helicobacter*. Culture: unclassified Chitinophagaceae strain, *Propionibacterium* sp.
The role of mucin cell-free DNA detection as a new marker for the study of acellular pseudomyxoma peritonei of appendicular origin by liquid biopsy	García-Olmo et al.	2020	Prospective laboratory study	n=2	Appendix	Low-grade PMP (n=2)	Mucin samples collected at the time of CRS	No comment	None	Unknown	16S metagenomic analysis	Phylum: Proteobacterias, Firmicutes, Actinobacteria. Class: Gammaproteobacterias, Alphaproteobacterias, Actinobacterias, Betaproteobacterias, Clostridia, Bacilli, Group II. Order: Peudomonadales, Sphingomonadales, Xanthomonadales, Actynomycetales, Rhizobiales, Burkholderiales, Clostridiales
Antibiotic treatment decreases microbial burden associated with pseudomyxoma peritonei and affects β-catenin distribution	Semino-mora et al.	2013	Prospective open-label study	n=48	Appendiceal mucinous neoplasms	Antibiotic cohort: Low-grade PMP (n=6), high-grade PMP (n=8). Control cohort: Low-grade PMP (n=13), high-grade PMP (n=21)	Tissue specimens collected at the time of CRS and HIPEC	No comment	Tissue from a patient with a nonperforated, nonneoplastic appendix (NNA) was used as a control	Control for nonspecific binding	ISH – probe hybridizes to 16S rRNA sequence conserved among 19,973 typed and nonculturable bacteria but not human sequences; probe specific for H.Pylori 16s rRNA. Parallel dual FISH studies were also conducted using the 16S rDNA-TNCB probe and the 16S rDNA-*H. pylori* probe	*H. pylori* and TNCB

Villarejo-Campos et al. and García-Olmo et al. conducted gene sequencing on fresh mucin samples prospectively collected from patients with PMP to identify and classify organisms at different taxonomic levels [27], 28]. Gilbreath et al. analysed archived formalin-fixed paraffin-embedded (FFPE) tissue specimens and mucin samples [29]. Several case studies identified previously unrelated or novel microorganisms from PMP samples. Gatalica et al. reported PMP associated with HPV-positive cervical cancer [30], whilst Lo et al. isolated a novel bacterial species, later re-classified by Lawson et al. [31], 32]. Another two studies examined the density of typed and non-culturable bacteria (TNCB), rather than identifying or classifying the organisms. Semino-Mora et al. compared the bacterial density of PMP samples from patients with no pre-operative antibiotic exposure to the density of non-neoplastic non-perforated appendix samples [33]. In a later study, Semino-Mora et al. prospectively examined the correlation between pre-operative antibiotics and the bacterial density of PMP samples, focussing on *H. pylori* [34].

In addition to the retrospective microbial analysis of PMP samples Gilbreath et al. also conducted a prospective case-control study comparing the survival of PMP patients that did and did not receive pre-operative antibiotics. The outcomes of these groups were updated five years later (Table 2) [35]. Whilst these two studies didn’t directly examine the PMP microbiome, they were developed to provide clinical context and guidance regarding the possible implications of their earlier results, and were therefore included for additional breadth.

**Table 2:** Summary of studies investigating effect of pre-operative antibiotics on patient survival.

Title	Author	Year	Follow-up period	Case cohort	Control cohort	PMP origin	Histology of antibiotic cohort	Lymph node status of antibiotic cohort	Histology of control cohort	Lymph note status of control cohort	Results
A core microbiome associated with the peritoneal tumours of pseudomyxoma peritonei	Gilbreath et al.	2013	65 months	Treated with lansoprazole + amoxicillin + clarithromycin (n=21)	n=37	Unknown	Low-grade PMP (n=8), high-grade PMP (n=11)	LN negative (n=13), LN positive (n=7), unknown (n=1). Distribution amonth low-grade and high-grade subsets unknown	High-grade PMP (n=37)	LN negative (n=37)	LN-negative high-grade PMP patients receiving antbiotics (n=6) were compared to LN-negative high-grade PMP patients without antibiotic exposure (n=37). The survival rate difference did not reach statistical significance but a higher proportion of the antibiotic cohort survived after 4.5 years (100 vs. ∼70 %)
Pre- and post-operative antibiotics in conjunction with cytoreductive surgery and heated intraperitoneal chemotherapy (HIPEC) should be considered for pseudomyxoma peritonei (PMP) treatment	Merrell et al.	2019	125 months	Treated with lansoprazole + amoxicillin + clarithromycin (n=17)	N/A	Unknown	Re-classification: low-grade PMP (n=6), high-grade PMP (n=11)	Low-grade PMP: unknown. High-grade PMP: LN-positive (n=4), LN-negative (n=7)	N/A	N/A	Outcomes of n=17 patients from antibiotic cohort updated after further five years. Low-grade PMP: lost to contact (n=1), alive with disease (n=1), alive without disease (n=4). High-grade PMP LN-positive: died of disease within 3.3 years (n=3), alive without disease (n=1). High-grade PMP LN-negative: died of disease (n=2), died of other causes (n=1), alive without disease (n=4). Outcomes of non-antibiotic cohort was not published

### Synthesis of results

#### Microorganisms identified in PMP

Villarejo-Campos et al. performed 16S rRNA gene sequencing and proteomic analysis by immunohistochemistry on mucin from six PMP patients [27]. Five patients had a primary low-grade appendiceal mucinous neoplasm (LAMN), and one had a primary mucinous colonic adenocarcinoma. Histological analysis of the specimens showed that of the patients with LAMN, two had low-grade PMP and three had acellular mucin. The patient with mucinous colonic adenocarcinoma had low-grade PMP. The mucin microbiome was characterised at the phylum level. Genomic DNA from *Proteobacteria* was the most prevalent in both acellular (82.68 %) and cellular (82.52 %) mucin, followed by Actinobacteria (8.17 % and 8.52 %, respectively) and Firmicutes (4.37 % and 4.56 %, respectively). For both types of mucin the most common bacterial order was *Pseudomonadales* (44 %) and the predominant genus was *Pseudomonas* (44 %). The authors went further to assess the viability and behaviour of the identified organism by inoculating both immunocompetent and immunocompromised mice with samples of the mucin. They found that the microbial community remained unchanged regardless of the host and immune status.

García-Olmo et al. also performed 16S sequencing on mucin collected during the CRS and HIPEC of two patients with low-grade PMP of appendiceal origin. Taxonomic analysis was carried out to identify the bacterial profile at phylum, class and order levels. The authors found that proteobacteria constituted the largest proportion of phyla (82.52–82.86 %), distantly followed by actinobacteria (8.17–8.52 %) and firmicutes (4.37–4.56 %). Among the proteobacteria, gammaproteobacterias were more prevalent than alphaproteobacterias, representing 52.8–59.41 % and 16.67–24.50 % of all bacteria identified at the class level. actinobacterias (7.16–8.51 %), betaproteobacterias (4.92–6.20 %), clostridia (1.47–2.27 %) and bacilli (2.04–3.05 %) represented a much lower proportion of the bacterial profile at the class level. For both patients, the order pseudomonadales was the most prevalent (44.55–45.00 %), with others including sphingomonadales (5.39–15.844 %) and xanthomonadales (7.36–14.16 %). The authors also injected human mucin into both immunocompetent and immunocompromised mice, and performed taxonomic analysis three weeks later, finding that the bacterial profile did not change. The authors did not attempt to culture the mucin bacteria, nor did they attempt to identify any microorganisms at the family, genus or species level.

Gilbreath et al. used V6 PCR and sequencing to profile the bacterial communities of paired tissue and mucin samples from 11 PMP patients who had not received antibiotics preoperatively [29]. Unfortunately, the primary tumour and the histological grade of each PMP specimen was not described. The microbial profiles were analysed first at the phylum level, and then at the genus level. The most prominent phylum represented in all samples was the proteobacteria, with a mean relative abundance of 73 %. Other prominent phyla included actinobacteria (mean relative abundance 10.7 %), firmicutes (6.9 %), bacteriodetes (7.2 %), verrucomicrobia (0.8 %), and acidobacteria (0.3 %). As the relative abundance of tumour and mucin microbiota did not differ significantly, the samples were combined for analysis at the genus level. This found that *Methylobacterium*, *Variovorax*, *Escherichia/Shigella* and *Pseudomonas* were the most prevalent proteobacteria. The authors also used 16S and 23S rRNA complementary probes to detect a subset of the taxa identified in the sequencing analysis, by ISH. Tissue section from three patients included in the sequencing analysis, plus two additional patients not previously included, were analysed. They confirmed the presence of *Pseudomonas, Propionibacterium* and *Streptococcus* sp in the PMP microbiome, although the relative density was not revealed. While neither the sequencing nor the ISH analysis confirmed the presence of viable and active microorganisms, the isolation and culture of organisms did. Eleven isolates from eight patients were analysed to that effect. The authors found active *Propionibacterium, Corynebacterium, Amycoliatopsis, Dermacoccus, Bosea* and *Niastella*, at the genus level. *Propionibacterium sp.* was most frequently isolated and was the only cultured taxon that had also been detected by ISH or genome sequencing.

Lo et al. continued the work of Gilbreath by culturing the bacteria from PMP tumour and mucin samples [31]. Genomic DNA was extracted for 16S rRNA sequencing. They identified a unique 16S sequence, PMP191F, which closely resembled a *Chitinophaga* species and a *Flavitalea populi* strain (HY-50R). Further analysis of the PMP191F genome revealed homology with *Chitinophaga pinensis* (90.3 %) and *Niastella koreensis* (84.7 %), although the 16S sequence-based phylogeny suggested a closer relationship with the latter. All results strongly suggested that PMP191F was a novel bacterial species. Lawson et al. extended these findings a few years later. They carried out and published extensive phylogenetic, phenotypic and chemotaxonomic analyses, demonstrating that PMP191F represents a novel species and genus within the family *Chitinophagaceae*. Given its similarity to members of the genus *Pseudoflavitalea*, they proposed the name *Parapseudoflavitalea muciniphila* gen. nov., sp. nov.

Semino-Mora et al. (2008) performed ISH on resection specimens to detect and quantify the density of TNCB including *H. pylori*, the virulence factor CagA, and the apomucins MUC2 and MUC5AC [33]. They compared the results of six low-grade PMP specimens and ten high-grade PMP specimens collected during CRS HIPEC procedures, to five non-neoplastic, non-perforated appendices (NNA) resected at the time of gynaecological procedures. Whilst enteric bacteria were detected in all specimens, bacterial density and MUC2 expression were significantly higher in high-grade PMP compared to low-grade PMP and controls. Interestingly, bacterial density was not significantly different in low-grade PMP vs. NNA. *H. pylori* was detected in all patients, but at a lower density than TNCB. The high-grade PMP had a significantly higher *H. pylori* density than low-grade PMP and NNA. The authors also observed that although MUC2 was detected in most specimens, the distribution was dependent on the histological grade of PMP. In low-grade PMP, higher levels of MUC2 were noted in the mucigen granules of goblet cells, the brush border of intestinal cells and in the free mucin, where it would be expected to be present in normal physiology. In high-grade PMP, however, MUC2 was also expressed in lymphoid inflammatory cells and in the stromal peri-vascular connective tissue. MUC2 expression was directly correlated with the density of TNCB in the epithelium, and with density of *H. pylori* in the epithelium and free mucin. Even though the data shows an association between the density of TNCB and *H. pylori* and the degree of mucin expression, particularly in higher histological grades of PMP, the evidence is insufficient to determine if the bacteria play a pathological role in this.

In 2013 Semino-Mora et al. published a prospective open-label study comparing the density of TNCB in the surgical specimens from 14 PMP patients who received preoperative triple therapy for *H. pylori* (lansoprazole, amoxicillin and clarithromycin) with that of 34 PMP patients who didn’t [33]. Tissue and mucin specimens were collected during CRS HIPEC performed one week after the completion of the antibiotic course. 16S rDNA probes capable of identifying 19,973 TNCB, as well as *H. pylori*, were used to quantify bacterial density through ISH. Laser confocal microscopy was also used to quantify cell membrane and nuclear localisation of β-catenin. The authors also found that *H. pylori* and TNCB densities were significantly higher in high-grade than in low-grade PMP. However, whilst *H. pylori* and TNCB densities were lower in high-grade PMP patients treated with antibiotics than in those who were not, there was no significant difference in the bacterial density among all low-grade PMP patients, regardless of antibiotic exposure. The authors also used polyclonal anti-*H. pylori* antibody to distinguish between viable and non-viable *H. Pylori*, demonstrating that the viability was reduced in the antibiotic-treated cohort. β-catenin expression was also higher in high-grade patients compared to the low-grade cohort. This suggests a relationship between the bacterial density and degree of β-catenin expression.

Gatalica et al. detected high-risk human papillomavirus in both the mucinous epithelium of the peritoneal tumour and the mucinous ascites fluid obtained intraoperatively [30]. Interestingly, this patient had undergone hysterectomy eight years prior for localised, well-differentiated mucinous adenocarcinoma of the cervix. Histological and immunophenotypic analysis also suggested that the intra-abdominal disease represented a metastatic recurrence of the primary endocervical adenocarcinoma.

#### Correlation between pre-operative antibiotics

Gilbreath et al. (2013) compared the outcomes of 21 PMP patients who received a 10 to 14-day course of amoxicillin, clarithromycin and lansoprazole prior to CRS HIPEC with those of 37 patients who received no antibiotics, over a period of five years [29]. Whilst the control group consisted only of lymph node negative high-grade PMP, the antibiotic group was more heterogeneous, consisting of both low-grade PMP (n=8), high-grade PMP (n=11), and one patient who had been diagnosed with both low-grade and high-grade PMP from separate biopsies. The antibiotic cohort also had varying lymph node status, with lymph node positive patients (n=13), lymph node negative patients (n=7) and one patient with unknown lymph node status. The authors found that lymph node negative patients who received preoperative antibiotics had a higher percentage survival rate than what was reported in the literature. However, the survival rates between high-grade PMP lymph node negative patients in the antibiotic group and the control group were not significantly different. Unfortunately, the survival analysis is rudimentary. The authors mainly describe the survival rates throughout a 4.5-year follow-up period with a Kaplan–Meier curve and did not collect sufficient data to perform multivariate analyses, which would have enabled a more robust interpretation of the results.

Five years later, Merrell et at. (2019) updated the survival rates of 17 patients from the antibiotic cohort [35]. They reported that two of the low-grade PMP patients were re-classified as high-grade PMP. Of low-grade PMP patients (n=6), four remained disease-free, one had relapsed, and another was lost to contact. Three of the four lymph node positive high-grade PMP patients died of disease within 3.3 years of the CRS and HIPEC. One lymph node positive high-grade PMP patient remained disease free after 10.4 years of follow-up. Of the lymph node negative high-grade PMP cohort (n=7), four remained alive without disease at the time of publication, two had died of disease and another had died of other causes. The exact disease-free survival and overall-survival rates of each cohort were not plotted against time. Additionally, the antibiotic-free cohort was not followed-up. Although the authors compared the outcomes of the antibiotic cohort with literature it would have been interesting to compare them to their own control cohort. Highlighting any further treatment received by patients particularly between time of relapse and time of death would have also allowed for a more comprehensive interpretation of their outcomes.

## Discussion

### Summary of evidence

Several organisms have been identified from PMP tissue or mucin samples (Table 3). Only two studies, Villarejo-Campos et al. and García-Olmo et al. used fresh tissue. A range of microbial detection and identification methods were used, including genomic sequencing, ISH and culture. At the phylum level, proteobacteria, was consistently detected in greatest relative abundance (73–82.86 %) in both PMP tumour tissue and both cellular and acellular mucin (Table 4). Other less abundant phyla included actinobacteriota, bacteroidetes/bacteroidota, firmicutes, verrucomicrobia and acidobacteria. Three studies detected *Pseudomonas* at the genus level, which alongside *Escherichia*, *Shigella*, *Methylobacterium* and *Variovorax* represented the most prevalent *Proteobacteria*.

**Table 3:** Microorganisms detected in PMP specimens by taxonomic rank.

Phylum	Class	Order	Family	Genus	Species
Proteobacteria SEQ^a,b,e^	Gammaproteobacteria ISH^a^ SEQ^e^	Xanthomonadales SEQ^b,e^	Xanthomonadaceae	*Stenotrophomonas* SEQ^b^	
		Enterobacterales	Enterobacteriaceae	*Escherichia-Shigella* SEQ^a^	
		Pseudomonadales SEQ^b,e^	Moraxellaceae	*Acinetobacter* SEQ^a^	
				*Moraxella* SEQ^a^	
			Pseudomonadaceae	*Pseudomonas* ISH^a^, SEQ^a,b^	*Pseudomonas plecoglossicida* SEQ^b^
	Betaproteobacteria ISH^a^ SEQ^e^	Burkholderiales SEQ^b,e^	Comamonadaceae	*Acidovorax* SEQ^a^	
				*Variovorax* SEQ^a^	
		Methylophilaceae	Methylophilaceae	*Methylotenera* SEQ^a^	
	Alphaproteobacteria SEQ^e^	Sphingomonadales SEQ^b,e^	Sphingomonadaceae	*Sphingomonas* SEQ^a^	
				*Sphingobium* SEQ^b^	
				*Novosphingobium* SEQ^b^	
		Rhizobiales SEQ^b,e^	Methylobacteriaceae	*Methylobacterium* ISH^a^, SEQ^a^ SEQ^b^	
			Boseaceae	*Bosea* CUL^a^	
Actinobacteriota ISH, SEQ^a,b,e^	Actinobacteria SEQ^e^	Pseudonocardiales	Pseudonocardiaceae SEQ^a^	*Saccharopolyspora* SEQ^b^	
				*Amycolatopsis* SEQ^a^	
		Corynebacteriales	Nocardiaceae	*Gordonia* SEQ^b^	
			Corynebacteriaceae	*Corynebacterium* CUL^a^	
		Propionibacteriales	*Propionibacteriaceae* SEQ^a^, CUL^a^	*Propionibacterium* SEQ^a^, CUL^a^, ISH^a^	
				*Tessaracoccus* SEQ^a^	
		Actinomycetales SEQ^b,e^			
Bacteroidetes ISH^a^, SEQ^a^	Bacteroidia	Chitinophagales	Chitinophagaceae	*Niastella* CUL^a^	*Parapseudoflavitalea muciniphila* gen. nov., sp. nov. CUL^c^
Firmicutes ISH^a^ SEQ^a,b,e^	Bacilli SEQ^e^	Lactobacillales	Streptococcaceae	*Streptococcus* ISH, SEQ^a^	
	Clostridia SEQ^e^	Eubacteriales	Clostridiaceae	*Clostridiales* SEQ^e^	
Verrucomicrobia ISH^a^, SEQ^a^	Verrucomicrobiia	Verrucomicrobiales ISHa			
Acidobacteria SEQ^a,e^					
Campylobacterota	Campylobacteria	Campylobacterales	Helicobacteraceae	*Helicobacter* ISH^a,d^	

Detection method: 16S ribosomal RNA sequencing (SEQ); Culture (CUL); *In situ* hybridization (ISH). Reference: ^a^Gilbreath et al., ^b^Villarejo-Campos al., ^c^Lawson et al., ^d^Semino-Mora et al., ^e^García-Olmo et al.

**Table 4:** Reported mean relative abundance (%) of bacteria in different types of PMP specimens detected through sequencing.

Phylum	Order	Genus
**Proteobacteria** Tissue and mucin: 73.0 %^a^ Acellular mucin: 82.86 %^b^, 82.69 %^e^ Cellular mucin: 82.52 %^b^	Xanthomonadales Acellular mucin: 7.36 %^b^, 10.76 %^e^ Cellular mucin: 14.16 %^b^	*Stenotrophomonas* Acellular mucin: 7.19 %^b^ cellular mucin: 13.11 %^b^
	Pseudomonadales Acellular mucin: 44.55 %^b^, 44.78 %^e^ Cellular mucin: 44.99 %^b^	*Pseudomonas* Acellular mucin: 44.90 %^b^ cellular mucin: 44.43 %^b^
	Burkholderiales Acellular mucin: 4.76 %^b^, 5.07 %^e^ Cellular mucin: 5.37 %^b^	
	Sphingomonadales Acellular mucin: 15.84 %^b^, 10.62 %^e^ Cellular mucin: 5.39 %^b^	*Sphingobium* Acellular mucin: *7.06* %^b^
		*Novosphingobium* Acellular mucin: *5.10* %^b^ Cellular mucin: *3.68* %^b^
	Rhizobiales Acellular mucin: 5.87 %^b^, 7.90 %^e^ Cellular mucin: 9.92 %^b^	*Methylobacterium* Acellular mucin: 4.89 %^b^ Cellular mucin: 7.87 %^b^
**Actinobacteria** Tissue and mucin: 10.7 %^a^ Acellular mucin: 8.17 %^b^, 8.35 %^e^ Cellular mucin: 8.52 %^b^		*Saccharopolyspora* Acellular mucin: 3.89 %^b^
		*Gordonia* Cellular mucin: 4.88 %^b^
	Actinomycetales Acellular mucin: 7.13 %^b^, 7.82 %^e^ Cellular mucin: 8.50 %^b^	
**Bacteroidetes** Tissue and mucin: 7.2 %^a^		
**Firmicutes** Tissue and mucin: 6.9 %^a^ Acellular mucin: 4.37 %^b^, 4.47 %^e^ Cellular mucin: 4.56 %^b^	Clostridiales Acellular mucin: 1.79 %^e^	
**Verrucomicrobia** Tissue and mucin: 0.8 %^a^		
**Acidobacteria** Tissue and mucin: 0.3 %^a^		

Reference: ^a^Gilbreath et al., ^b^Vilarejo et al., ^e^García-Olmo et al.

Interestingly, in the healthy human gut a higher proportion of firmicutes, bacteroidetes and actinobacteria is seen and proteobacteria represent only a small minority of the total metagenomic species [36], [37], [38]. Whereas in inflammatory bowel disease, a reduction in firmicutes and rise in proteobacteria has been reported [39], 40], which is more in-keeping with the microbiome of PMP specimens. The difference in oxygen levels may contribute to this shift in phylogenetic profile [41], 42].

Some of the genera identified in PMP specimens, like *Stenotrophomonas*, *Escherichia*, *Shigella*, *Moraxella*, *Pseudomonas*, *Streptococcus* and *Helicobacter*, include species that exist on the spectrum of microbe-host relationships, from commensal organisms to pathogens. The relevance of these organisms in the progression or outcome of PMP is uncertain. Others, such as *Saccharopolyspora*, *Sphingobium*, *Methylotenera*, *Variovorax* and *Acidovorax,* have seldom been associated with human disease are more commonly seen in environments like soil, marine sediments, and plants, thus are more likely to represent contaminants.

The histological grade of PMP, on the other hand, is a strong prognostic factor, with high-grade PMP conferring worse survival than low-grade PMP [43]. High-grade specimens showed significantly higher bacterial density, *H. pylori* inclusive, than low-grade specimens and non-neoplastic non-perforated appendix specimens [33], 34]. However, the bacterial densities from the latter two cohorts were not significantly different. It is unclear whether the increased bacterial density is contributing towards the increased cytological atypia and mitotic activity seen in high-grade PMP or whether they simply thrive better in that environment. Therefore, histological grade may be a major confounding factor in the effect of bacterial density on disease outcome.

Two interesting relationships which may explain this association have been highlighted. One is between bacterial density and MUC2 expression and the other between bacterial density and β-catenin expression.

The MUC genes transcribe the glycoprotein that form mucin. MUC2 is expressed by healthy goblet cells in the intestine but is overexpressed in gastric and colorectal cancers [44], 45]. Semino-Mora et al. [33] observed that both bacterial density and MUC2 expression were significantly higher in high-grade PMP than in low-grade PMP. Previous work had evidenced the transcriptional activation of mucin by *Pseudomonas aeruginosa* lipopolysaccharide in cystic fibrosis [46] and PMP [47], suggesting higher bacterial densities, particularly of species such as *P. aeruginosa*, may be contributing to increased MUC2 expression and therefore volume of peritoneal mucin. While there may be a link between certain bacteria and MUC2 overexpression in addition to an independent correlation between the histological grade of PMP and total MUC2 expression, it is difficult to draw a link between all three factors. This is due to evidence suggesting that the increased MUC2 expression in high-grade PMP is due to a greater number of mucinous tumour cells rather than gene overexpression at a cellular level [47]. Perhaps then, the bacterial density could be attributed to the cellularity of the mucin, which is greater in high-grade PMP than low-grade PMP, resulting a richer environment for growth, although there is currently little evidence to support this theory.

It has also been suggested that bacteria may modulate β-catenin signalling pathways [48], 49], which are known to regulate apoptosis as well as the transcription of oncogenes. Semino-Mora et al. observed that β-catenin expression was higher in high-grade PMP specimens compared to the low-grade PMP specimens, although the difference was not statistically significant [34]. The authors also demonstrated that pre-operative antibiotics were associated with significantly lower β-catenin expression in high-grade PMP specimens, but not in low-grade specimens. Whilst they claimed that the antibiotics were responsible for this decrease, they didn’t perform a longitudinal analysis before and after antibiotic treatment, nor did they account for other confounders. Although the correlation between bacterial density, β-catenin and histological grade was observed in this small sample size, a causative effect has not been proven.

Even though the data shows an association between the density of TNCB and *H. pylori* and the degree of mucin expression, particularly in higher histological grades of PMP, the evidence is insufficient to determine if the bacteria play a pathological role in this.

The impact of pre-operative antibiotics survival on PMP patients was also investigated. Initially, Semino-mora et al. demonstrated that antimicrobial treatment was associated with reduced *H. pylori* and TNCB densities [34]. Subsequently, they followed-up the patient from the antibiotic cohort over a ten-year period and reported their outcomes. The relevance of these results is severely limited by our inability to compare them to the non-antibiotic cohort, as well as the small sample size. Furthermore, the authors did not record or acknowledge confounding factors which may have influence survival, nor did they perform any statistical analysis, particularly multivariate, which would have increased the interpretability of their results. More robust survival studies are required to consolidate our understanding of the potential role of antibiotics in the management of PMP.

### Limitations of existing evidence

A significant limitation of all studies included was the small cohorts of PMP patients included, which is unsurprising given the rarity of the condition. Additionally, there was considerable variation in the type of specimen analysed (i.e. fresh tissue, FFPE, mucin), the molecular detection methods, and PMP tumour origin. This hindered our ability to compare the results and draw meaningful conclusion.

Furthermore, there was often no comment on confounding factors, such as unintentional pre-operative antibiotic exposure or evidence of contamination during specimen collection. Nor was there rigorous use of controls. Experts have raised concerns about the impact of contamination, not only during clinical collection procedures but also during the extraction and sequencing process, and cautioned about the risk of that confounding the results [50]. In fact, contamination could be responsible for the detection of bacteria commonly associated with nosocomial infections, such as *Sphingomonas* and *Bosea*, in several of the studies discussed in this review [27], 29].

Better understanding of the limitations of detection methods is also required to avoid drawing erroneous conclusions. While detection methods such as genome sequencing has greatly enhanced the ability to detect microorganisms, they cannot discern viability cross-sectionally [51], 52]. Traditionally, culture and isolation has been used to identify viable organisms, although more recent strategies like viability PCR have been developed [53], 54]. Only two studies attempted to do this, one through culture [29], and another using a polyclonal anti-*H. pylori* antibody to discern the viability of the identified *H. pylori* [34]. Without evidence of organism viability, the ability to determine their role in this disease is significantly limited [29].

### Suggested direction for future study

Multicentre studies, involving specialist centres, may be able to overcome the issue of small sample size by pooling together their patients to compile larger cohorts. Clear description of the tumour origin and histology, type of specimen collected, and possible confounding factors such as antibiotic exposure or evidence of contamination during the collection process, is crucial for greater comparability of results.

Current studies lack species-level resolution, which can be difficult to achieve when intratumoral microbial biomass is low. Optimising methodology will be key to pinpoint potentially oncogenic organisms or clusters. This can be achieved through the use of controls to assess contaminant DNA and microbial detection methods that provide higher resolution profiling [55], [56], [57].

Ultraclean DNA extraction kit and concurrent sequencing of negative control samples, for example, may reduce contamination by the ‘kitome’ [58], [59], [60]. The use of paraffin controls in addition to FFPE tissues would also enable authors to discount bacterial communities originating from the paraffin itself [61]. Furthermore, comparing the tumour bacterial profile with the patient’s own gut microbiome would lend valuable insight, by highlighting differences in bacterial profiles or conversely providing further evidence of surgical contamination.

Using detection methods that are capable of discerning viable organisms from non-viable ones, such as viability PCR and culture, would clarify the role of microorganism in carcinogenesis or disease progression. However, this can only be confirmed with evidence of the exact mechanism of action, given the wide spectrum of microbe-host interactions. Examining PMP tumour cells or cellular mucin *in vitro* alongside specific microorganism in a stromal environment, such as with MUC2-secreting human intestinal cell lines, could provide a new useful research model.

### Review limitations

Only three databases were used as part of our search strategy. Although a large number of results were generated, only a few of these were relevant. Given the specificity of our search question, we could have performed forward and backward reference searching to maximise relevant results. Our review was also limited by the small number of screeners and reviewers (n=2), resulting in higher risk of selection bias. There was some missing data identified during the extraction process, including tumour origin and tissue type, which affected the interpretation and comparability of results.

### Conclusions

This review aimed to identify what organisms have been detected in PMP specimens, and correlate that with evidence of pathogenicity and effect on disease outcome.

Several bacteria were identified from tumour and mucin specimens, at different taxonomic ranks. The relative proportion of different phyla more closely resembles the microbiome seen in inflammatory bowel disease than in the healthy gut, with a higher relative abundance of Proteobacteria compared to Firmicutes and Bacteroidetes. Only *Pseudomonas plecoglossicida* and a novel bacterium, *P. muciniphila gen. nov., sp. nov.,* were identified at the species level. Most of the organisms described were identified through molecular sequencing, so their viability is questionable. However, bacteria from the genera *Bosea, Corynebacterium, Propionibacterium* and *Niastella* were successfully cultured from PMP samples. Whilst there may be a tenable association between bacterial density and histological grade, the nature of this relationship is unclear.

There is insufficient evidence to establish a causal link between the microbial alterations observed and any tumour behaviour or clinical outcomes. No definitive conclusions can be made regarding the microbiota’s role in PMP progression.

Significant methodological challenges remain in this field of study. Ongoing research, using considered and cautious methodology, has the potential to catapult our understanding of this malignancy and change the landscape of its clinical management.

## Appendix 1: PRISMA-ScR checklist.

**Table:**

Section	Item	PRISMA-ScR checklist item	Reported on page #
**Title**			

Title	1	Identify the report as a scoping review	1

**Abstract**			

Structured summary	2	Provide a structured summary that includes (as applicable): background, objectives, eligibility criteria, sources of evidence, charting methods, results, and conclusions that relate to the review questions and objectives	2

**Introduction**			

Rationale	3	Describe the rationale for the review in the context of what is already known. Explain why the review questions/objectives lend themselves to a scoping review approach	3
Objectives	4	Provide an explicit statement of the questions and objectives being addressed with reference to their key elements (e.g., population or participants, concepts, and context) or other relevant key elements used to conceptualize the review questions and/or objectives	4

**Methods**			

Protocol and registration	5	Indicate whether a review protocol exists; state if and where it can be accessed (e.g., a Web address); and if available, provide registration information, including the registration number	5
Eligibility criteria	6	Specify characteristics of the sources of evidence used as eligibility criteria (e.g., years considered, language, and publication status), and provide a rationale	5
Information sources	7	Describe all information sources in the search (e.g., databases with dates of coverage and contact with authors to identify additional sources), as well as the date the most recent search was executed	5
Search	8	Present the full electronic search strategy for at least 1 database, including any limits used, such that it could be repeated	5
Selection of sources of evidence	9	State the process for selecting sources of evidence (i.e., screening and eligibility) included in the scoping review	6
Data charting process	10	Describe the methods of charting data from the included sources of evidence (e.g., calibrated forms or forms that have been tested by the team before their use, and whether data charting was done independently or in duplicate) and any processes for obtaining and confirming data from investigators.	6
Data items	11	List and define all variables for which data were sought and any assumptions and simplifications made	6
Critical appraisal of individual sources of evidence	12	If done, provide a rationale for conducting a critical appraisal of included sources of evidence; describe the methods used and how this information was used in any data synthesis (if appropriate)	N/A
Synthesis of results	13	Describe the methods of handling and summarizing the data that were charted	6

**Results**			

Selection of sources of evidence	14	Give numbers of sources of evidence screened, assessed for eligibility, and included in the review, with reasons for exclusions at each stage, ideally using a flow diagram	7
Characteristics of sources of evidence	15	For each source of evidence, present characteristics for which data were charted and provide the citations	7
Critical appraisal within sources of evidence	16	If done, present data on critical appraisal of included sources of evidence (see item 12)	N/A
Results of individual sources of evidence	17	For each included source of evidence, present the relevant data that were charted that relate to the review questions and objectives	28–29
Synthesis of results	18	Summarize and/or present the charting results as they relate to the review questions and objectives	8

**Discussion**			

Summary of evidence	19	Summarize the main results (including an overview of concepts, themes, and types of evidence available), link to the review questions and objectives, and consider the relevance to key groups	14
Limitations	20	Discuss the limitations of the scoping review process	18
Conclusions	21	Provide a general interpretation of the results with respect to the review questions and objectives, as well as potential implications and/or next steps	19

**Funding**			

Funding	22	Describe sources of funding for the included sources of evidence, as well as sources of funding for the scoping review. Describe the role of the funders of the scoping review	19

Tricco AC, Lillie E, Zarin W, O’Brien KK, Colquhoun H, Levac D, et al. PRISMA Extension for Scoping Reviews (PRISMAScR): checklist and explanation. Ann Intern Med. 2018;169:467–473. doi: 10.7326/M18-0850.
